# Utility of telemedicine in pediatric rheumatology during the COVID-19 pandemic

**DOI:** 10.1186/s12969-021-00624-z

**Published:** 2021-09-30

**Authors:** Ashley Perdue, Charles Mullett, Amna Umer, Paul Rosen

**Affiliations:** 1 Department of Pediatrics, West Virginia University, Morgantown, WV USA; 2 West Virginia University School of Medicine, Morgantown, WV USA; 3 Department of Pediatrics/Research, West Virginia University, Morgantown, WV USA

**Keywords:** Pediatric, Rheumatology, COVID-19, Telemedicine, Telehealth

## Abstract

**Background:**

During the COVID-19 pandemic, telemedicine has provided an alternative to in-person visits for patients practicing social distancing and undergoing quarantine. During this time, there has been a rapid expansion of telemedicine and its implementation in various clinical specialties and settings. In this observational study we aim to examine the utility of telemedicine in a pediatric rheumatology clinic, for 3 months during the COVID-19 pandemic.

**Methods:**

A review of outpatient pediatric rheumatology telemedicine encounters were conducted from April–June 2020. Telemedicine visits (*n* = 75) were compared to patients seen in practice over the prior year in office-based visits (March 2019–March 2020) (*n* = 415). Patient characteristics, information on no-show visits, completed visits, new patient or follow-up status, and if new patients had received a visit within 2 weeks of calling to schedule an appointment were analyzed by chart review. An independent sample t-test and Chi Square statistic was used to determine statical significance between the two groups. A two-proportion z-test was used to compare visit metrics.

**Results:**

The percentage of new patients utilizing telemedicine (60%) was lower and statistically significant compared to the percentage of new patient office visits (84%) the previous year (*p* < 0.0001). There was no change in no-show rate between groups and patient characteristics were similar.

**Conclusions:**

This study demonstrates a statistically significant decrease in new patient visits during the pandemic with telemedicine-only appointments compared to in-office visits over the previous year. This suggests a possible hesitation to seek care during this time. However, there was no significant difference among patient characteristics between telemedicine visits during the pandemic and during in-office visits in the previous year. In our experience, patient visits were able to be conducted via telemedicine with a limited physical exam using caregiver’s help during the pandemic. However, further studies will need to ascertain patient satisfaction and preference for telemedicine in the future.

## Introduction

The coronavirus disease 2019 (COVID-19) was declared a pandemic by the World Health Organization (WHO) on the 11th of March 2020 [1]. Since the pandemic started, there has been a rise in the usage of telemedicine. Telemedicine provides an alternative way to care for patients who are social distancing and/or undergoing quarantine. It provides a substitute to office visits for patients, and limits exposure, while preserving personal protective equipment and avoiding patient travel. The use of smartphones and tablets makes real-time audio-visual communication easily accessible to both physicians and their patients. However, the application of telemedicine can be limited by broadband access in rural and underserved communities.

Telemedicine prior to the COVID-19 pandemic had begun to grow in the United States but was not widespread. Telemedicine or tele-visit usage doubled in use from 2016 to 2019 [2]. However, while telemedicine has grown in recent years it was still under-utilized. A 2019 survey on telehealth reported that only 8% of consumers had participated in a video visit with a doctor [3]. In both general and subspecialty pediatrics, telemedicine was under-utilized before the pandemic. In pediatric rheumatology, in 2014, only seven pediatric rheumatology clinics had telemedicine capability, with only three clinics ever reporting using it [4]. Considering there are several states without a pediatric rheumatologist, there are patients who could benefit from telemedicine services. However, many barriers to telemedicine still exist, such as concerns about privacy, preference for in-person care, and provider reimbursement [3].]. Telemedicine can also be a barrier for those who have out-of-state patients due to licensing, however through the Interstate Medical Licensure Compact that currently includes thirty states, the District of Columbia, and the Territory of Guam, physicians can practice in multiple states [5].

Telemedicine has been used successfully in a variety of ways across specialties before the COVID-19 pandemic. Telemedicine has been described previously in subspecialties of pediatrics such as pediatric psychiatry, pediatric cardiology, and neonatology [6–8]. Telemedicine with videoconferencing has previously been successful with rural populations for chronic disease management allowing for increased access and convenience [9]. Aside from use in rural settings, telemedicine has been described for disaster relief. In 2017, Nemours Children’s Hospital in Orlando, Florida used pediatric telemedicine during Hurricane Irma [10]. This implies that telemedicine can be used when access is limited during a public emergency, which has been seen as the trend with telemedicine usage during the pandemic this past year.

The Covid-19 pandemic has sparked a rapid expansion of telemedicine and its implementation in various clinical specialties and medical settings. Telemedicine is an alternative to in-person visits when patients may be wary of coming to a medical office during a pandemic. During the Covid-19 pandemic the number of ambulatory visits declined nearly 60% by early April 2020. Simultaneously, telemedicine visits increased rapidly to 14% of visits over the month of April [11]. While there has been a recent rebound in in-person visits, the recovery has been lowest among certain specialties including sub-specialty pediatrics, with school-age children showing the smallest number of rebound visits [11]. This lack of rebound in visits may be due to caregivers’ continued precaution to travel to clinics and potentially expose their children to the virus. This illustrates the importance of how telemedicine can be utilized in sub-specialty pediatrics, such as pediatric rheumatology during the pandemic.

Prior to the pandemic, there were few studies on the use of telemedicine in pediatric rheumatology. In one pre-pandemic survey, 95% of families with pediatric rheumatology patients reported they had a preference for in-person visits over telemedicine [12]. Interestingly, this preference was similar regardless of patient viewpoint on travel being inconvenient or convenient to the clinic [12]. While the previous survey found that patients had a preference for in-person visits, another study found that telemedicine-based clinics in pediatric rheumatology have statistically significant decreases in median distance traveled to clinic, amount of money spent on food while traveling, and less time missed from work and school when compared to in office encounters [13].

It is important to understand how best to utilize telemedicine in a post-pandemic healthcare setting. With the recent expansion of telemedicine use, lessons learned on how to integrate a virtual platform into practice should be shared. This study is the first study comparing patient populations who utilized telemedicine in pediatric rheumatology during the COVID-19 pandemic to patient populations with office-visits in the prior year. In our academic pediatric rheumatology clinic, visits were changed from all office based to all telehealth in April 2020. We report our three-month experience with telehealth in pediatric rheumatology during the period of April–June 2020.

## Methods

### Telemedicine

A review of outpatient telehealth encounters was conducted in an academic pediatric rheumatology practice. Patient encounters were conducted via telehealth only from April–June 2020. These visits were compared to patients seen in the practice over the prior year in office-based visits. The prior year of in-office based visits was used as a comparison as the pediatric rheumatology clinic started as of March of 2019 at West Virginia University. The clinic was the first dedicated pediatric rheumatology practice in the history of the State of West Virginia. The clinic was staffed by a board-certified pediatric rheumatologist who completed pediatric rheumatology training in 2003. The clinic was staffed at a 50% full time equivalent (FTE) level. The pediatric rheumatologist lived 3 states away. Telemedicine was offered to provide care in the absence of in-office visits due to travel restrictions in effect. Telemedicine visits were only offered during April–June 2020 and patients did not have an alternative option during this time as the clinic was otherwise closed during the early months of the pandemic.

In order for patients to participate in telehealth visits patients were given an instructional video before their appointment on how to download and access the MyChart Epic Health Systems app on their smartphone or tablet. Once the app was downloaded patients accepted a user agreement and logged in with their MyChart account. Patients had an E-check-in where they consented to a telemedicine visit. Patients were then able to access their video appointment through the MyChart app using video and audio linking themselves and the physician. For patients who could not connect, or did not have video capabilities, audio only was used for the appointment. The physician completed general telemedicine courses for formal training, however, experience overtime using telemedicine helped improve physical exam skills on the telemedicine platform. During telehealth-only visits from April to June of 2020 patients were located in their own homes during the visit. Patients had access to a University-based technology helpline if they had difficulty accessing the telehealth visit. If patients forgot or missed an appointment the appointment would be rescheduled for a future time. There were no limitations to telemedicine visits and any patient could participate. Patients were not pre-screened in any way based on diagnosis, new vs. follow-up patient, or disease severity. All patients were offered a telemedicine visit during this time. The number of appointment slots, as well as time interval for appointments, remained the same for telemedicine appointments as they were the prior year for in-office visits.

During telehealth visits, the physician met the patient and family and documented the history. A physical exam was then performed by visualization. The adult caregiver was an active participant by helping to point the camera on the specific joints or to help bend the joints for the physician. An assessment was provided and orders for laboratory tests, imaging, or referrals were entered as well as any necessary prescriptions. The family was able to ask any questions and were provided the doctor’s contact information for any follow-up questions.

### Chart review

Patient characteristics were analyzed by chart review in the EPIC electronic medical records (EMR), system comparing office visits (conducted March 2019–March 2020) and telehealth visits (conducted April–June 2020). The chart review included pediatric patients who had made appointments with the pediatric rheumatology outpatient practice. Charts were analyzed to obtain information on no-show visits, completed visits, new patient or follow-up status, and if new patients had received a visit within 2 weeks of calling to schedule an appointment. Charts were also reviewed for patient characteristics such as age, diagnosis, and zip code.

From March 2019 to March 2020 (pre-pandemic), there were 415 unique patients identified who completed office visits. One patient was excluded from analysis because of an invalid home address. Two patients were excluded from analysis because the address listed was several states away, making them outliers. Only unique patient visits were included; repeat visits were not included in order to avoid duplicate counting of demographic information. Patients could be included in both groups once if they had an office visit in the prior year and had completed a telemedicine visit during the April–June 2020 timeframe.

From April–June 2020 (during the pandemic), 75 unique patients were identified who completed telemedicine visits. No in-office visits were completed during this time. Two visits were conducted by audio only, due to limited internet bandwidth. One visit was conducted with a nurse practitioner, with the patient located in a rural clinic site close to the patient’s home.

Patient zip codes were obtained from the chart review and used to estimate average household income. Patient addresses in the chart were used to calculate miles from the clinic to determine distance traveled. To determine if patients lived in a medically underserved area (MUA) their zip codes were entered into the “Am I Rural?” tool from the Rural Health Information Hub to determine their rural status. This tool is supported by the United States Health Resources and Services Administration (HRSA) and the United States Department of Health and Human Services (HHS) [14].

The information obtained from telehealth visits during April–June 2020 was then compared to the information obtained from in-office visits from the previous year, March 2019 – March 2020, to examine if there was a statistically significant difference in the population of patients utilizing telemedicine compared to in-office visits during the COVID-19 pandemic. The study was approved by the institutional review board of West Virginia University.

#### Statistical analysis

An independent samples t-test was used to determine statistically significant difference between the means of the two groups (office visit pre-pandemic and tele-visit during pandemic) for the continuous variables. The continuous variables included patient’s age, distance from the clinic, and annual income. The Chi Square statistic was used to determine whether there is a statistically significant association between the two groups (office visit pre-pandemic and tele-visit during pandemic) and categorical variables. The categorical variables included rural designation by zip code, juvenile idiopathic arthritis diagnosis, and pediatric fibromyalgia diagnosis. A two-proportion z-test was used to examine the difference between two population proportions for data available in aggregate form (% new patients, new patient visits < 2 weeks, and no-show rate). Alpha was set at 0.05 for all statistical tests.

## Results

Patient characteristics comparing office visits to telemedicine visits are shown in Table 1. For telemedicine encounters, 97% used both video and audio.

**Table 1:** Comparison of patient characteristic between telemedicine and office visits

	Office visits March 2019–March 2020		Telemedicine visits April 2020–June 2020		*p*-value
	Mean / Percentage (*N* = 415)	SD	Mean / Percentage (*N* = 72)	SD	
Mean patient age (years)	11.71	4.63	11.94	4.87	0.6951
Mean distance from clinic (miles)	95.71	88.74	100.3	72.83	0.6759
Mean annual income by zip code (dollars)	$55,556	12,643.1	$57,195	12,358.1	0.3088
Rural designation by zip code (%)	51.57%		52.78%		0.8504
Diagnosis: juvenile idiopathic arthritis (%)	13.73%		29.17%		0.001
Diagnosis: pediatric fibromyalgia (%)	24.10%		22.22%		0.7304

Independent sample t-test for continuous variables

Chi-square test for categorical variables

The average age of patients utilizing office visits and telemedicine visits showed no statistical difference. The average age of patients using telemedicine was 11.71 years with an age range of 2 to 19 years. The average age for office visits was 11.94 years with an age range of 1 to 21 years. Both groups of patients lived similar distances away from the clinic with telemedicine visits living a mean distance of 100.3 miles and in-office visits living 95.71 miles. Rural designation and mean annual income were not significantly different as well, with telemedicine being 52.78% rural and in-office visits being 51.57% rural. Mean annual family income was $57,195 for the telemedicine group and $55,556 for the in-office group. There were 47 diagnoses seen over the previous year of in-office visits and 16 diagnoses seen during telemedicine only encounters for the 3 months into the pandemic. The diagnoses can be seen in Table 2. The most common diagnoses seen with the telemedicine visits were juvenile idiopathic arthritis (JIA), followed by pediatric fibromyalgia. A statistically significantly higher percentage of children with JIA were seen in telemedicine visits (29.17% *p* = 0.001) compared to in-office visits the previous year (13.73%). The percentage of fibromyalgia patients were not significantly different for office visits and telemedicine visits, 24.10 and 22.22% respectively. The most common diagnosis seen in the office the previous year was fibromyalgia (24.10%) followed by JIA (13.73%).

**Table 2:** Diagnoses seen during Telemedicine and In-Office Visits

In-Office Visits (March 2019–March 2020) (n)	Telemedicine Visits (April 2020–June 2020) (n)
Alopecia (1)	Chronic Osteomyelitis (1)
Amplified Musculoskeletal Pain Syndrome (3)	Chronic Vasculitis (1)
Chiari I Malformation (1)	Cutaneous Lupus (1)
Chondromalacia (6)	Fever of Unknown Origin (2)
Chronic Fatigue Syndrome (3)	Fibromyalgia (17)
Chronic Intractable Headache (1)	Granulomatosis with Polyangiitis (1)
Chronic Osteomyelitis (3)	Juvenile Dermatomyositis (1)
Complex Regional Pain Syndrome (6)	Juvenile Idiopathic Arthritis (22)
Cutaneous Lupus Erythematosus (1)	Lyme Arthritis (2)
Ehlers-Danlos Syndrome (6)	Mixed Connective Tissue Disease (1)
Erythema Multiforme (1)	Periodic Fever, Aphthous Stomatitis, Pharyngitis, Adenitis (1)
Erythema Nodosum (1)	Psoriasis (3)
Failure to Thrive (1)	Raynaud’s Without Gangrene (1)
Fever of Unknown Origin (2)	Reactive Arthritis (1)
Fever, Unspecified Cause (5)	Reflex Sympathetic Dystrophy (1)
Fibromyalgia (100)	Uveitis (1)
Flat Feet (1)	
Glomerulonephritis (2)	
Gout (1)	
Granulomatosis with Polyangiitis (1)	
Hashimoto Encephalopathy (1)	
Hashimoto’s Disease (1)	
Henoch-Schonlein Purpura (4)	
Hypogammaglobulinemia (1)	
Intermittent Fever of Unknown Origin (2)	
Juvenile Dermatomyositis (1)	
Juvenile Idiopathic Arthritis (57)	
Lichen Sclerosis (1)	
Lyme Arthritis (2)	
Lyme Disease (1)	
Lupus (1)	
Mediterranean Fever (1)	
Nephrotic Syndrome (1)	
Patella-Femoral Pain Syndrome (1)	
Parotitis (1)	
Pediatric Autoimmune Neuropsychiatric Disorders Associated with Streptococcus (1)	
Periodic Fever Syndrome (10)	
Postural Orthostatic Tachycardia Syndrome (2)	
Psoriasis (2)	
Psoriatic Arthritis (3)	
Raynaud’s Without Gangrene (15)	
Reactive Arthritis (3)	
Reflex Sympathetic Dystrophy (2)	
Scleroderma (1)	
Sjogren’s Syndrome (1)	
Streptococcal Arthritis (1)	
Uveitis (2)	

Practice metrics comparing the two different time frames are shown in Table 3. The percentage of new patients utilizing telemedicine was lower and statistically significant compared to the percentage of new patient office visits (*p* < 0.0001). During the time of telehealth only visits for the first 3 months into the pandemic there were 60% new patients, while there were 84% new patients for in-office visits during the one pre-pandemic year. The percentage of patients who received an appointment within 2 weeks of calling for a new consultation for office visits and telemedicine was similar at 61 and 69.30% respectively. The no-show rate for office visits and telemedicine were not significantly different at 6 and 6.7% respectively.

**Table 3:** Comparison of encounters between telehealth and office visits

Table 2	Office visits March 2019–March 2020	Telehealth visits April 2020–June 2020	z-test to compare two proportion
Number of patients (N)	415	75	
% new patients	83%	60%	< 0.0001
New patient visits < 2 weeks	61%	69.30%	0.1723
No show rate	6%	6.70%	0.2525

## Discussion

This is the first study to compare patients who utilized telemedicine for pediatric rheumatology during the public health emergency caused by the COVID-19 to patients who had in-office visits in the prior year. We found no change in no show rates with telemedicine compared to office visits. Percentage of new office visits in the office setting decreased from 83 to 60% in the telemedicine setting. This decrease in new patient visits could be due to various reasons. One potential reason could be that families decided to defer care during the beginning of the pandemic all together. This could also be due to the decrease in healthcare utilization that occurred during the beginning of the pandemic that was seen across all specialties nationwide. Another potential reason could be that families did not believe telemedicine would be able to address their concerns. Families might have also been uncomfortable with trying to use telemedicine and instead deferred care until in-office visits resumed. Follow-up patients also may have been more likely to utilize telemedicine during this time because of their previously established doctor-patient relationship. Family preferences regarding telemedicine during the pandemic will require further study. Based on patient demographics such as distance from clinic, family’s annual income, patient age, and rural status, patients utilizing telemedicine during the pandemic were similar to those using office visits before the pandemic. The pandemic may have made families more amenable to trying telemedicine for the first time for their children with pediatric rheumatology concerns. However, families may have used telemedicine during this time out of necessity as in-office visits were not available, which could explain why demographics and patient characteristics were not significantly different compared to in-office visits over the previous year pre-pandemic.

This study found that there was a statistically significant increase in JIA visits during telemedicine compared to in-office visits in the previous year. The JIA patient follow-up schedule is different than that for fibromyalgia. This may have led to more telemedicine follow-up visits for JIA. Also, JIA patients who were already established with the physician may have been more amendable to using telemedicine during this time. New vs follow-up JIA telemedicine patient characteristics were not explored due to too low of a patient sample size.

When utilizing telemedicine, the physician was still able to assess patient response to treatment. The physician could assess response to treatment and verify diagnosis with serial visualization on telemedicine exams, and history reported by family. In-office follow-up visits were also made available once the pandemic started to wane. Starting in July 2020 the clinic reverted back to office visits; however, the template was changed to accommodate both in-office and telemedicine visits. Patients, families and the pediatric rheumatologist used a shared-decision making approach to decide the venue of follow-up visits. Families were able to choose telemedicine, office visits, or a mix of both. Areas for future study in pediatric rheumatology include: family preference for telemedicine vs. office visits and family satisfaction with telemedicine visits.

With telemedicine visits offered during the COVID-19 pandemic, we were able to garner experience with its benefits and limitations. One advantage is that telemedicine allows for small children to be relaxed during the exam in the confines of their own home. Physicians can make a game out of the exam over audio-video communication. This setting also allows the physician the ability to get a general sense of the patient’s home environment when visits are conducted from the patient’s kitchen or living room. The downside to using audio-video communication is some limitations of the physical exam. An important aspect of the pediatric rheumatology exam is an examination of the joints including palpation, which can be done indirectly by training the caregiver how to assist. The telemedicine exam facilitates the physician to coach the caregiver as an assistant and makes the caregiver an active participant in the exam. The caregiver can help position the child, move the joints, and report the child’s pain level. In this way, the family has a more active role during the child’s visit.

The pediatric rheumatology group at Stanford Children’s has begun a multiphase pilot study that examines the clinical gap in video visits. From their preliminary data they have found that the rheumatologic physical exam, vital signs, modes of communication between provider and patient, need for immediate laboratory work or imaging, and the need for nursing or social work support are all barriers to the adoption of telemedicine when compared to the in-person clinic visits [15]. This describes the several barriers that physicians and patients encounter on the telemedicine platform that will need to be addressed.

In addition to some of the limitations that telemedicine can create, physicians will also have to become comfortable with the lack of formality telemedicine can engender. In our experience, some families arrived to their telemedicine visits in pajamas. Other visits were conducted with the child in the car with the parent driving. Clearer instructions about what to expect during the telemedicine visit should be given to families ahead of the visits. The patient-physician relationship has to be established on the screen. Optimizing this virtual relationship is something both patients and physicians will have to learn going forward together.

Technology barriers to telemedicine also remain. Patients are required to learn how to use the technology in order to participate in a telemedicine visit. There must be appropriate access to technology and internet for patients to participate, which in rural areas may be limited. Families may also have devices without camera capabilities, limiting their ability to utilize video communication further hindering the ability to try to conduct a physical exam virtually. It will be important to assess a family’s ability and comfort utilizing technology prior to a telemedicine visit. Patients in the future could also benefit from an instructional video on how telemedicine visits are conducted in order to set expectations and to show how the platform is operated. In our study, clinic patients were provided an instructional video prior to their visit on how to operate the telemedicine platform.

A recent article in *Pediatric Rheumatology* discussed the potential glitches with telehealth utilization during the COVID-19 pandemic, with one potential limitation being the inability to ask pediatric patients about psychosocial matters such as depression and smoking while a parent may be in the room or helping to operate the telehealth visit [16]. This poses another challenge with telehealth for providers who need to assess important psychosocial factors that may contribute to their patient’s disease or their compliance with treatment. This is important in pediatric rheumatology as many diagnoses are chronic and psychosocial factors should be assessed. Healthcare professionals will have to learn how to navigate these conversations over a screen in order to maintain the patient-provider relationship and the patient’s confidentiality.

A recent study in an Alaskan population assessed the outcomes and quality of care for rheumatoid arthritis in telemedicine. They found that telemedicine did not improve rheumatoid arthritis activity or quality of care over a 12-month period, however it was found that it was not inferior to in-person care. Perceptions of telemedicine were also addressed using a telemedicine perception survey. They found that individuals using telemedicine still expressed a preference to be seen in-person, however those using telemedicine were more likely to feel that the care given was as good as in-person visits. Both telemedicine patients and office visits patients felt that technical difficulties were a limitation to its use [17]. This study suggests that telemedicine may offer another option of care to patients that is as good as in-person visits. This study also shows that those utilizing telemedicine have more positive perceptions about its use as an alternative method of care.

Further studies will need to ascertain patient satisfaction with telemedicine in pediatric rheumatology. The growth of telemedicine during the COVID-19 pandemic has rapidly transformed the way providers deliver care. Some families may feel more comfortable with telemedicine after trying it for the first time. Other families may feel most comfortable in the office setting only. It would also be important to understand why some patients may have deferred care during the pandemic and whether it was due to their perceptions about telemedicine. Exploring family preferences for modality of care will be an important area for future study.

Some valuable lessons were learned during the first 3 months of our telemedicine experience. Beyond the pandemic, telemedicine will have a place in pediatric rheumatology care where access issues persist. With less than 500 pediatric rheumatologists in the United States, there are still nine states lacking access to pediatric rheumatology care. If telehealth could be utilized further, then patient access could be enhanced across the country. This would include rural areas, underserved areas, and areas where families have to travel long distances to receive care. The Covid-19 pandemic is an event that will stimulate physicians to evaluate how they can expand care within a small subspecialty. More families may choose to try a telemedicine visit if given the option. The pediatric rheumatology workforce should be ready to embrace telemedicine as part of the future of serving the patient population. Learning a digital physical exam and interacting with families over video will be the new normal in many instances. How to best leverage technology for the care of pediatric rheumatology patients requires further study. Next steps will be to study the accuracy of telemedicine diagnostics versus in-person visits, patient choice regarding utilizing telemedicine, as well as patient satisfaction with telemedicine. In the spring of 2021 patient volume of the clinic had recovered to pre-pandemic numbers and both in-office and telemedicine visits were offered.

### Limitations

In this study, only one board certified pediatric rheumatologist was included. The practice at the institution has one pediatric rheumatologist who sees all patients and therefore all diagnoses and experiences included are limited to one physician. In-office visits were used from March 2019–March 2020 and compared to the first 3 months period of telemedicine visits during the COVID-19 pandemic (April 2020–June 2020). Since there were months included in the previous year of appointments that were not included during the pandemic there could be possible confounders such as but not limited to seasonality and school schedule. We did not study diagnostic accuracy between in-office visits and telemedicine visits. However, we did learn of the utility of telemedicine in pediatric rheumatology care during a public health emergency. When using telemedicine visits you are losing the touch proponent of the physical exam and must instead rely on visual inspection, range of motion, or the instruction of a parent to feel. While this study did not explore diagnostic accuracy, this study does show that in time of crisis telemedicine can be used in conjunction with office visits for care. The severity of disease among patients cannot be commented on in this study. Practicing behavior, number of resources used, or number of labs ordered was not compared in this study, therefore we cannot comment on the difference in care received. Patients using telemedicine were not administered patient satisfaction surveys. Patient satisfaction with telemedicine was not accessed with this study. This study was conducted on a population of patients mostly residing in West Virginia. Patients in our area also included Western Maryland, Eastern Ohio, and Southwestern Pennsylvania.

## Conclusions

This study found that there was a statistically significant decrease in the number of new-patient visits during telemedicine-only visits during the COVID-19 pandemic from April–June 2020 when compared to office visits in the previous year. This study also reports no significant change in no-show rate or patient characteristics during telemedicine in April–June 2020 when compared to in-office visits the previous year. This is the first study to compare patient characteristics and office visit metrics in a pediatric rheumatology practice during the COVID-19 pandemic with telemedicine usage. Now that the pandemic has accelerated the use of telemedicine, further study should evaluate patient attitudes, preferences, and satisfaction. Additional study among providers should assess diagnostic error rates, resource utilization, prescribing patterns, and the patient-provider relationship. Telemedicine is another tool in the pediatric rheumatologist toolkit to provide access to care for families seeking care within a small subspecialty with a limited supply of providers in any given geographic area.

## Data Availability

The datasets used and/or analyzed during the current study are available from the corresponding author on reasonable request.

## Abbreviations

COVID-19: Corona Virus Disease 2019
WHO: World Health Organization
EMR: Electronic Medical Records
MUA: Medically Underserved Area
HRSA: United States Health Resources and Services Administration
HHS: United States Department of Health and Human Services
JIA: Juvenile Idiopathic Arthritis
